# Effectiveness of Screening Using Fecal Occult Blood Testing and Colonoscopy on the Risk of Colorectal Cancer: The Japan Public Health Center-based Prospective Study

**DOI:** 10.2188/jea.JE20210057

**Published:** 2023-02-05

**Authors:** Kenta Tanaka, Tomotaka Sobue, Ling Zha, Tetsuhisa Kitamura, Norie Sawada, Motoki Iwasaki, Manami Inoue, Taiki Yamaji, Shoichiro Tsugane

**Affiliations:** 1 Department of Social and Environmental Medicine, Graduate School of Medicine, Osaka University, Osaka, Japan; 2 Epidemiology and Prevention Group, Center for Public Health Sciences, National Cancer Center, Tokyo, Japan

**Keywords:** colorectal cancer, colorectal cancer screening, fecal occult blood test, colonoscopy, prospective cohort study

## Abstract

**Background:**

Few cohort studies have used multiple surveys of screening attendance to simultaneously evaluate the effectiveness of fecal occult blood test (FOBT) and colonoscopy.

**Methods:**

We analyzed data of 30,381 middle-aged Japanese adults from a population-based prospective cohort study. Information on FOBT and colonoscopy was obtained from three questionnaire surveys (every 5 years). We classified the subjects into three groups: the FOBT (15,649 subjects), screening colonoscopy (2,407 subjects), and unscreened (12,325 subjects) groups. We used the unscreened group as the reference group to compare the mortality and incidence of colorectal cancer (CRC).

**Results:**

During the 14-year follow-up, 64, 12, and 104 CRC deaths were identified in the FOBT, screening colonoscopy, and unscreened groups, respectively. The risk of CRC death reduced with increasing the number of FOBTs (*P* for trend = 0.02) and was reduced by 44% in the subjects screened twice or thrice using FOBT (hazard ratio [HR] 0.56; 95% confidence interval [CI], 0.33–0.94). Significant decreases were seen for the incidence of CRC but not seen for the incidence of non-advanced CRC in the FOBT group. Concerning the screening colonoscopy, subjects screened at the start of follow-up showed a 69% reduced risk of CRC death (HR 0.31; 95% CI, 0.10–0.9996). Significant decreases were also seen for the incidence of CRC and non-advanced CRC in the subjects screened at the start of follow-up.

**Conclusion:**

FOBT, depending on the number of FOBTs, and colonoscopy, depending on recency, reduced the risk of death due to CRC and the incidence of CRC.

## INTRODUCTION

Colorectal cancer (CRC) was the second leading cause of cancer-related death and the third most common cancer worldwide in 2018.^1^ Cancer screening is a way to decrease the number of deaths due to CRC. The United States Preventive Services Task Force and the American Cancer Society have recently recommended guaiac-based fecal occult blood testing and fecal immunochemical testing every year, flexible sigmoidoscopy every 5 years, and colonoscopy every 10 years for CRC screening.^2^^,^^3^ In Japan, screening programs using annual fecal immunochemical tests (FITs) have been implemented nationwide since 1992.^4^ According to the 2005 guidelines for CRC screening, the fecal occult blood test (FOBT) is recommended for organized and opportunistic (grade A) screenings, and colonoscopy is recommended only for opportunistic (grade C) screening.^5^

These recommendations are based on accumulated studies, including randomized controlled trials (RCTs), case-control studies, and cohort studies. Cohort studies regarding the effectiveness of cancer screening often use information on one-time exposure, such as the previous Japan Public Health Center-based prospective study (JPHC) on the efficacy of FOBT,^6^ or divide the exposure into two (screened and unscreened). In addition, limited studies have assessed the effectiveness of FOBT and colonoscopy simultaneously in reducing the incidence of CRC and risk of death due to CRC.

Therefore, this study aimed to evaluate the effectiveness of FOBT and colonoscopy in reducing mortality associated with CRC using information on multiple exposures in the Japanese population from a large-scale population-based prospective cohort study with a 14-year follow-up period.

## METHODS

### Settings

We used data from the JPHC cohort I, which included subjects aged 40–59 years and was started in January 1990. The cohort areas included five prefectural public health centers (PHCs): Iwate (Ninohe), Akita (Yokote), Nagano (Saku), Okinawa (Chubu), and Tokyo (Katsushika). The study design has been described in detail elsewhere.^7^^,^^8^ Self-administered questionnaires were administered at baseline (Q00), 5-year follow-up (Q05), and 10-year follow-up (Q10) to collect comprehensive information, including the sociodemographic characteristics, medical history, screening experience, diet, and other lifestyle-related factors. The study protocol was approved by the institutional review boards of the National Cancer Center, Tokyo, Japan and Osaka University, Osaka, Japan. Individuals who responded to the questionnaire were considered to have consented to participate in the study.

In our analysis, the Tokyo area was excluded because its study population was defined differently from the others and incidence data on cancer cases were not available.

### Study population

**Figure 1.**
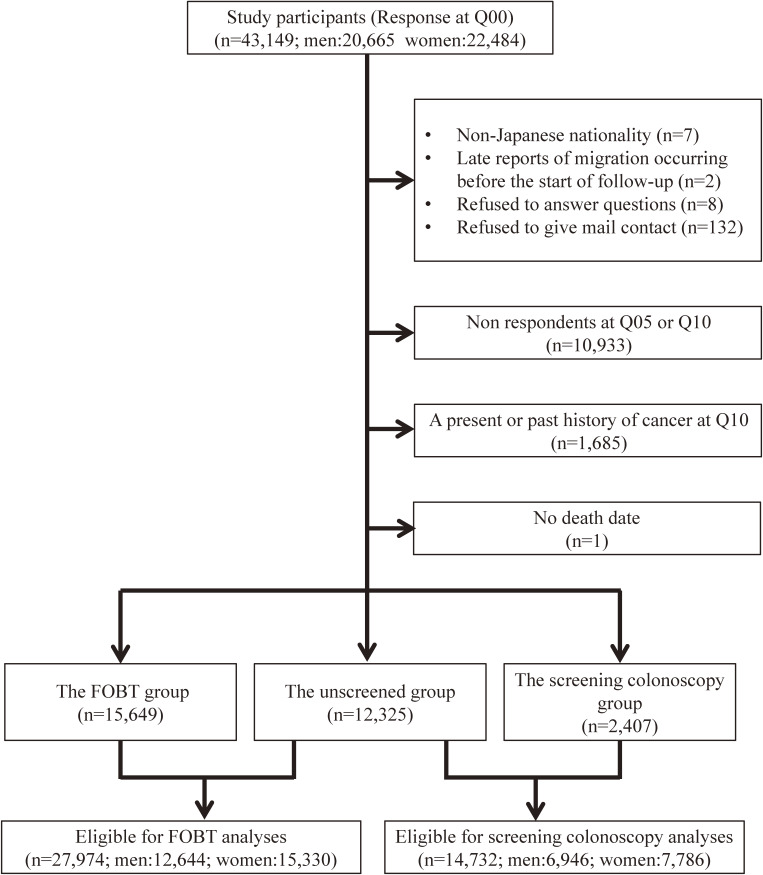
Subject flowchart. FOBT, fecal occult blood test.

The study population comprised all registered Japanese inhabitants in 14 administrative districts, supervised by four PHC areas. The Japanese inhabitants were identified from population registries maintained by local municipalities. In the baseline survey, 43,149 subjects (20,665 men and 22,484 women) responded (Figure 1). Subjects who were not Japanese nationals (*n* = 7), with late reports of migration occurring before the start of the study (*n* = 2), those who refused to participate (*n* = 8), and those who refused to provide their mail contact (*n* = 132) were excluded. Further, we excluded subjects who did not respond at Q05 or Q10 (*n* = 10,933), those with a present or past history of any cancer at Q10 (*n* = 1,685) using questionnaire and registry data, and those without a death date (*n* = 1). Ultimately, 30,381 subjects were included in our analyses.

### Exposure definition

Next, we obtained information on FOBT and colonoscopy based on the response provided by the subjects regarding screening experience in the self-administered questionnaire: yes (screening performed during 1 year preceding Q00, Q05, and Q10) and no. For this study, we divided colonoscopy into diagnostic and screening colonoscopy. When the subjects answered in the same questionnaire that colonoscopy was performed and FOBT was not performed, this colonoscopy was classified as screening colonoscopy. However, when the subjects answered in the same questionnaire that both colonoscopy and FOBT were performed, this colonoscopy was classified as diagnostic colonoscopy, assuming that it was conducted after a positive FOBT result. Furthermore, we divided the subjects into three groups: those who underwent screening colonoscopy at least once at Q00, Q05, or Q10 were classified in the screening colonoscopy group (*n* = 2,407); those who underwent at least one FOBT but no screening colonoscopy were classified in the FOBT group (*n* = 15,649); and those who did not undergo CRC screening were classified in the unscreened group (*n* = 12,325). For the analysis of FOBT, we compared the FOBT group with the unscreened group. For the analysis of screening colonoscopy, we compared the screening colonoscopy group with the unscreened group. Finally, 27,974 eligible subjects were included in the FOBT analysis, and 14,732 eligible subjects were included in the screening colonoscopy analysis (Figure 1).

### Follow-up

The follow-up period was from the date of response to Q10 until December 31, 2013. Therefore, Q10 was used as the start of follow-up in this analysis. Follow-up information on residence status and vital status was collected using the residential registry.^8^ Because the Family Registration Law in Japan mandates registration of deaths, the registry has complete information. Information on the cause of death was supplemented with ascertainment of death certificate files provided after permission was obtained from the Ministry of Health, Labour and Welfare. The causes of death were classified according to the International Classification of Diseases (ICD), 10th Revision, as follows: deaths from CRC (C18–C20) and all cancers (C00–C97).^9^

The occurrence of cancer was identified using local hospital records and population-based cancer registries in the study area, with permission from each of the local governments responsible for the cancer registries. Death certificate information was referred to as a supplementary information source. Cancer site and histology were classified according to the ICD for Oncology, 3rd Edition (ICD-O-3), as follows: CRC (C18–C20) and all cancers (C00–C97).^10^

For the analysis of risk of death due to CRC, person-years were determined from the date of the Q10 survey until the date of death or the end of the study period (December 31, 2013), whichever occurred first. For the analysis of incidence of CRC, person-years were determined from the date of the Q10 survey until the date of CRC diagnosis, the date of emigration from the study area, the date of death, or the end of the study period, whichever occurred first. For subjects who were lost to follow-up, the last confirmed date of their presence in the study area was used as the censor date.

### Outcomes

The primary outcome was death from CRC. To assess potential selection bias, death from all cancers excluding CRC, all causes of death excluding CRC, incidence of CRC, and all cancer incidence excluding CRC were used as secondary outcome variables. For further investigation, we divided CRC cases into advanced and non-advanced cases. In cases in which CRC was invading the muscularis propria or deeper (T2–T4 in the Tumor, Node, Metastasis Classification) were defined as advanced cancer cases.^11^

### Statistical analysis

We collected the history of FOBT and colonoscopy three times (Q00, Q05, and Q10), and categorized the history of FOBT by the number of FOBTs and the history of screening colonoscopy by recency. For the analysis of FOBT, the screening history was classified into three categories (0, 1, and 2–3 times) according to the number of FOBTs. For the analysis of screening colonoscopy, the screening history was classified into four categories (the start of follow-up [Q10], 5 years before the start of follow-up [Q05], 10 years before the start of follow-up [Q00], and unscreened) according to the latest screening colonoscopy.

Differences in proportions and mean values for potential confounding factors among each of the categories were assessed. Hazard ratios (HRs) and 95% confidence intervals (CIs) were estimated using the Cox proportional hazards regression model to assess the relationship of the risk of death due to CRC and the incidence of CRC with CRC screening. We adjusted for age at Q10 (continuous), sex, study area (four PHC areas), and potential confounding factors, including smoking status (never, former, current, or missing); alcohol intake (none, occasional, regular, or missing); history of diabetes (yes or no); body mass index; physical activity (metabolic equivalents); occupation (working, unemployed or homemaker, or unknown); and energy-adjusted red and processed meat, vegetable, fish, fruit, dairy product, and coffee intakes (quintiles). We referred to the World Cancer Research Fund (https://www.wcrf.org/dietandcancer/colorectal-cancer) for diet, nutrition, physical activity, and CRC. Additionally, we adjusted for screening histories of chest (0–1, 2, or 3 times) and stomach (0, 1, or 2–3 times) X-ray at Q00, Q05, and Q10 (the number of chest and stomach X-ray) considering selection bias. Significance tests were two sided, and *P*-values <0.05 were considered statistically significant. Statistical analyses were performed using Stata, version 13.1 (Stata Corp, College Station, TX, USA).

## RESULTS

During the 14-year follow-up, in the FOBT group (15,649 subjects; 6,879 men and 8,770 women), 64 CRC deaths and 391 CRC cases were identified. In the screening colonoscopy group (2,407 subjects; 1,181 men and 1,226 women), 12 CRC deaths and 46 CRC cases were identified. In the unscreened group (12,325 subjects; 5,765 men and 6,560 women), 104 CRC deaths and 409 CRC cases were identified.

The characteristics of the study subjects at the start of follow-up in the FOBT analysis are shown in Table 1. The number of the screened subjects (once, and twice or thrice) were 7,793 and 7,856, respectively. Subjects who had undergone more FOBTs had a lower body mass index; were more likely to be never smokers and regular alcohol drinkers; were less likely to report a history of diabetes; consumed less red and processed meat and coffee; consumed more vegetable, fish, fruit, and dairy products; had more physical activity; and underwent more chest and stomach X-rays than those who had undergone no or few FOBTs. The characteristics of the study subjects at the start of follow-up in the analysis of the screening colonoscopy group are shown in Table 2. The number of screened subjects at 10 years before the start of follow-up, 5 years before the start of follow-up, and at the start of follow-up were 284, 714, and 1,409, respectively. Subjects who had undergone screening colonoscopy were more likely to have a history of diabetes and underwent more chest and stomach X-rays than those who had not undergone screening colonoscopy. Further, their eating habits were similar to subjects who had undergone more FOBTs.

**Table 1.** Characteristics of study subjects at the start of follow-up according to the number of FOBTs

	0	1	2 or 3
	(*n* = 12,325)	(*n* = 7,793)	(*n* = 7,856)
Proportion, %	44.06	27.86	28.08
Sex, %			
Male	46.77	44.33	43.58
Female	53.23	55.67	56.42

Age, years	59.2 (5.9)	59.7 (5.9)	59.9 (5.8)

Body mass index, kg/m^2^	23.9 (3.2)	23.8 (3.1)	23.7 (2.9)

Smoking status, %			
Never	63.76	66.61	68.94
Former	12.16	13.01	14.50
Current	24.08	20.38	16.56

Alcohol drinking status, %			
None	53.76	53.13	51.37
Occasional	7.57	8.11	8.20
Regular	38.67	38.76	40.43

History of diabetes, %			
No	93.91	94.71	95.33
Yes	6.09	5.29	4.67

Occupation, %			
Working	57.92	57.58	60.09
Unemployed or homemaker	37.93	39.63	38.43
Unknown	4.15	2.80	1.48

Red and processed meat intake, g/day	44.0 (41.9)	41.2 (37.2)	39.3 (33.1)

Vegetable intake, g/day	236.9 (166.8)	256.2 (166.0)	280.0 (170.8)

Fish intake, g/day	75.8 (60.7)	78.6 (56.6)	79.4 (50.5)

Fruit intake, g/day	190.9 (181.2)	207.4 (183.5)	220.6 (170.6)

Dairy product intake, g/day	175.6 (205.4)	192.1 (202.3)	206.3 (193.1)

Coffee intake, g/day	135.1 (192.9)	124.3 (174.6)	108.7 (157.4)

Physical activity, MET-h/day	40.9 (7.9)	41.1 (7.7)	41.6 (7.5)

Number of chest X-rays, %			
0 or 1	39.96	15.09	2.41
2	29.05	32.27	18.89
3	30.99	52.64	78.70

Number of stomach X-rays, %			
0	61.92	29.10	15.99
1	23.38	34.45	22.05
2 or 3	14.69	36.44	61.97

FOBT, fecal occult blood test; MET-h, metabolic equivalent task hours.

Values reported as mean (standard deviation) unless otherwise noted.

**Table 2.** Characteristics of study subjects at the start of follow-up according to the latest screening colonoscopy

	Unscreened	10 years before the start of follow-up	5 years before the start of follow-up	The start of follow-up
	(*n* = 12,325)	(*n* = 284)	(*n* = 714)	(*n* = 1,409)
Proportion, %	83.66	1.93	4.85	9.56
Sex, %				
Male	46.77	48.59	45.38	51.03
Female	53.23	51.41	54.62	48.97

Age, years^a^	59.2 (5.9)	60.6 (5.9)	60.4 (5.7)	60.6 (5.7)

Body mass index, kg/m^2 a^	23.9 (3.2)	23.6 (3.1)	23.8 (3.1)	23.8 (3.0)

Smoking status, %				
Never	63.76	60.99	69.17	62.40
Former	12.16	18.44	12.16	16.15
Current	24.08	20.57	18.67	21.44

Alcohol drinking status, %				
None	53.76	52.30	51.78	48.03
Occasional	7.57	8.83	7.13	7.65
Regular	38.67	38.87	41.08	44.32

History of diabetes, %				
No	93.91	94.01	92.86	92.12
Yes	6.09	5.99	7.14	7.88

Occupation, %				
Working	57.92	60.21	54.90	59.90
Unemployed or homemaker	37.93	37.32	41.60	38.47
Unknown	4.15	2.46	3.50	1.63

Red and processed meat intake, g/day^a^	44.0 (41.9)	38.2 (33.9)	41.5 (37.4)	40.4 (36.4)

Vegetable intake, g/day^a^	237.1 (166.8)	273.1 (180.6)	259.2 (187.0)	252.7 (165.8)

Fish intake, g/day^a^	75.9 (60.7)	74.8 (47.4)	78.5 (60.5)	77.6 (60.2)

Fruit intake, g/day^a^	191.0 (181.1)	205.7 (169.2)	199.7 (174.6)	208.9 (189.1)

Dairy product intake, g/day^a^	175.6 (205.3)	198.9 (201.9)	216.3 (253.9)	198.8 (221.8)

Coffee intake, g/day^a^	135.2 (193.1)	99.4 (142.3)	115.2 (172.8)	101.8 (160.3)

Physical activity, MET-h/day^a^	40.9 (7.9)	41.1 (7.7)	41.0 (7.8)	41.2 (7.8)

Number of chest X-rays, %				
0 or 1	39.96	23.59	17.37	13.20
2	29.05	29.58	30.53	27.04
3	30.99	46.83	52.10	59.76

Number of stomach X-rays, %				
0	61.92	25.70	27.73	24.98
1	23.38	35.21	33.19	31.01
2 or 3	14.69	39.08	39.08	44.00

MET-h, metabolic equivalent task hours.

^a^Mean (standard deviation).

The HRs for risk of CRC according to the number of FOBTs are shown in Table 3. In subjects screened once and twice/thrice using FOBT, the risk of death due to CRC was reduced with an increase in the number of FOBTs, and the risk was significantly reduced by 44% in the subjects screened twice/thrice using FOBT (HR 0.56; 95% CI, 0.33–0.94; *P* for trend = 0.02). Reductions in the risk of all-cause death excluding CRC were observed in the screened subjects. The degree of risk reduction of death due to CRC was greater than that due to all other causes. The incidence of CRC in subjects screened once and twice/thrice decreased with an increase in the number of FOBTs (HR 0.85; 95% CI, 0.71–1.02 and HR 0.68; 95% CI, 0.55–0.85, respectively; *P* for trend <0.01). Risk reduction was seen in advanced CRC, but not in non-advanced CRC. There was no significant reduction in the risk of all cancers excluding CRC.

**Table 3.** Hazard ratio and 95% confidence interval for the number of FOBTs and subsequent risk of colorectal cancer in 27,974 subjects in the JPHC Study

	Number of FOBTs			
	0	1	2 or 3	*P* for trend
Death				
Person-years of follow-up	162,124.6	104,173.4	106,506.5	
Colorectal cancer				
Number of deaths (*n* = 168)	104	38	26	
HR (95% CI)^a^	1.00 (reference)	0.58 (0.40–0.85)	0.42 (0.27–0.65)	<0.01
HR (95% CI)^b^	1.00 (reference)	0.75 (0.50–1.12)	0.56 (0.33–0.94)	0.02

All cancers excluding colorectal cancer				
Number of deaths (*n* = 1,161)	606	316	239	
HR (95% CI)^a^	1.00 (reference)	0.82 (0.71–0.94)	0.65 (0.55–0.76)	<0.01
HR (95% CI)^b^	1.00 (reference)	0.88 (0.76–1.02)	0.74 (0.62–0.89)	<0.01

All causes of death excluding colorectal cancer				
Number of deaths (*n* = 3,191)	1,683	868	640	
HR (95% CI)^a^	1.00 (reference)	0.82 (0.76–0.89)	0.64 (0.58–0.70)	<0.01
HR (95% CI)^b^	1.00 (reference)	0.90 (0.82–0.98)	0.77 (0.69–0.86)	<0.01

Incidence				
Person-years of follow-up	157,735.6	101,733.2	104,334.3	
Colorectal cancer				
Number of cases (*n* = 800)	409	218	173	
HR (95% CI)^a^	1.00 (reference)	0.81 (0.68–0.95)	0.66 (0.54–0.79)	<0.01
HR (95% CI)^b^	1.00 (reference)	0.85 (0.71–1.02)	0.68 (0.55–0.85)	<0.01

Advanced colorectal cancer				
Number of cases (*n* = 410)	236	91	83	
HR (95% CI)^a^	1.00 (reference)	0.57 (0.45–0.73)	0.51 (0.39–0.66)	<0.01
HR (95% CI)^b^	1.00 (reference)	0.65 (0.50–0.84)	0.58 (0.43–0.79)	<0.01

Non-advanced colorectal cancer				
Number of cases (*n* = 307)	130	101	76	
HR (95% CI)^a^	1.00 (reference)	1.18 (0.91–1.54)	0.94 (0.70–1.27)	0.86
HR (95% CI)^b^	1.00 (reference)	1.16 (0.87–1.54)	0.86 (0.61–1.22)	0.49

All cancers excluding colorectal cancer				
Person-years of follow-up	155,206.7	99,262.9	100,912.8	
Number of cases (*n* = 3,087)	1,305	869	913	
HR (95% CI)^a^	1.00 (reference)	1.03 (0.95–1.13)	1.06 (0.97–1.16)	0.17
HR (95% CI)^b^	1.00 (reference)	1.02 (0.93–1.12)	1.05 (0.95–1.17)	0.33

CI, confidence interval; FOBT, fecal occult blood test; HR, hazard ratio.

^a^Adjusted for age at Q10, sex, study area, smoking status, alcohol drinking status, history of diabetes, occupation, body mass index, physical activity, red and processed meat intake, vegetable intake, fish intake, fruit intake, diary intake, and coffee intake.

^b^Additionally adjusted for the number of chest and stomach X-rays.

The HRs for the risk of CRC according to screening colonoscopy are shown in Table 4. The subjects screened at the start of follow-up showed a 69% reduced risk of death due to CRC compared with unscreened subjects (HR 0.31; 95% CI, 0.10–0.9996), but there was no reduction among the subjects screened 10 years before the start of follow-up and 5 years before the start of follow-up. The incidence of CRC decreased significantly in subjects screened at the start of follow-up (HR 0.40; 95% CI, 0.26–0.64), but did not decrease significantly in those screened 10 years and 5 years before the start of follow-up. Decreases in the incidence of advanced CRC were seen in subjects screened 5 years before the start of follow-up and at the start of follow-up. Decreases in the incidence of non-advanced CRC were seen in subjects screened at the start of follow-up. Regarding the incidence of CRC, the *P* for trend was significant. There was no significant reduction in the risk of all cancers excluding CRC.

**Table 4.** Hazard ratio and 95% confidence interval for the recency of screening colonoscopy and subsequent risk of colorectal cancer in 14,732 subjects in the JPHC Study

	The recency of screening colonoscopy				
	Unscreened	10 years before the start of follow-up	5 years before the start of follow-up	The start of follow-up	*P* for trend
Death					
Person-years of follow-up	162,124.6	3,684.8	9,362.9	18,597.6	
Colorectal cancer					
Number of deaths (*n* = 116)	104	3	6	3	
HR (95% CI)^a^	1.00 (reference)	1.18 (0.37–3.77)	0.96 (0.42–2.20)	0.24 (0.07–0.75)	0.02
HR (95% CI)^b^	1.00 (reference)	1.50 (0.46–4.85)	1.24 (0.53–2.88)	0.31 (0.10–0.9996)	0.13

All cancers excluding colorectal cancer					
Number of deaths (*n* = 722)	606	13	31	72	
HR (95% CI)^a^	1.00 (reference)	0.83 (0.48–1.44)	0.84 (0.58–1.20)	0.90 (0.70–1.15)	0.24
HR (95% CI)^b^	1.00 (reference)	0.88 (0.50–1.53)	0.89 (0.62–1.28)	0.95 (0.73–1.23)	0.57

All causes of death excluding colorectal cancer					
Number of deaths (*n* = 2,018)	1,683	45	99	191	
HR (95% CI)^a^	1.00 (reference)	1.05 (0.78–1.41)	0.98 (0.80–1.20)	0.88 (0.75–1.02)	0.12
HR (95% CI)^b^	1.00 (reference)	1.13 (0.84–1.53)	1.05 (0.85–1.29)	0.96 (0.82–1.12)	0.79

Incidence					
Person-years of follow-up	157,735.6	3,594.3	9,204.1	18,260.9	
Colorectal cancer					
Number of cases (*n* = 455)	409	10	16	20	
HR (95% CI)^a^	1.00 (reference)	0.96 (0.51–1.81)	0.62 (0.38–1.03)	0.38 (0.24–0.59)	<0.01
HR (95% CI)^b^	1.00 (reference)	1.03 (0.54–1.94)	0.66 (0.40–1.10)	0.40 (0.26–0.64)	<0.01

Advanced colorectal cancer					
Number of cases (*n* = 260)	236	5	7	12	
HR (95% CI)^a^	1.00 (reference)	0.77 (0.32–1.89)	0.45 (0.21–0.96)	0.38 (0.21–0.68)	<0.01
HR (95% CI)^b^	1.00 (reference)	0.90 (0.36–2.20)	0.52 (0.24–1.12)	0.44 (0.24–0.81)	<0.01

Non-advanced colorectal cancer					
Number of cases (*n* = 148)	130	4	7	7	
HR (95% CI)^a^	1.00 (reference)	1.30 (0.48–3.55)	0.85 (0.40–1.84)	0.41 (0.19–0.87)	0.03
HR (95% CI)^b^	1.00 (reference)	1.24 (0.45–3.42)	0.83 (0.38–1.80)	0.39 (0.18–0.85)	0.02

All cancers excluding colorectal cancer					
Person-years of follow-up	155,206.7	3,554.0	8,907.5	17,541.6	
Number of cases (*n* = 1,620)	1,305	30	88	197	
HR (95% CI)^a^	1.00 (reference)	0.89 (0.61–1.27)	1.10 (0.89–1.37)	1.16 (0.998–1.35)	0.047
HR (95% CI)^b^	1.00 (reference)	0.84 (0.58–1.22)	1.06 (0.85–1.32)	1.11 (0.95–1.30)	0.20

CI, confidence interval; HR, hazard ratio.

^a^Adjusted for age at Q10, sex, study area, smoking status, alcohol drinking status, history of diabetes, occupation, body mass index, physical activity, red and processed meat intake, vegetable intake, fish intake, fruit intake, diary intake, and coffee intake.

^b^Additionally adjusted for the number of chest and stomach X-rays.

In total, 40, 4, and 43 cases were unclassified into advanced or non-advanced cases in the FOBT, screening colonoscopy, and unscreened groups, respectively; these were excluded from the analysis of incidence of advanced and non-advanced CRC.

The HRs for risk of CRC according to the recency of FOBT are shown in eTable 1. The tendency was similar with the recency of screening colonoscopy; however, the degree of reduction of the risks appeared to be less than that of screening colonoscopy. Even if we analyzed the recency of screening colonoscopy and FOBT limited in subjects screened once, the results did not change substantially (data not shown).

## DISCUSSION

This population-based prospective study evaluated the impact of the history of CRC screening on the mortality and incidence of CRC. During the 14-year follow-up, this study revealed that FOBT, depending on the number of FOBTs, and colonoscopy, depending on recency, reduced the risk of death due to CRC and the incidence of CRC.

For the analysis of FOBT, we categorized the subjects according to the number of FOBTs and for the analysis of screening colonoscopy, we categorized the subjects according to the recency of screening colonoscopy. In this study, the proportion of subjects who underwent FOBT twice or thrice was about 28%. It is possible that the subjects underwent FOBT during the follow-up period. In this study, about 74% of those who underwent FOBT at both Q00 and Q05 underwent FOBT at Q10, and about 75% of those who did not undergo FOBT at either Q00 or Q05 did not undergo FOBT at Q10. The number of FOBTs in the past more accurately estimates the situation of undergoing FOBT during the follow-up period than the latest FOBT alone; hence, we used the number of FOBTs. In contrast, the proportion of subjects who underwent screening colonoscopy once a year was <5%, and that of subjects who underwent screening colonoscopy thrice was approximately 0.5%. We considered that there were few screened subjects who underwent screening colonoscopy during the follow-up period; hence, we evaluated the latest screening colonoscopy.

In the analysis of FOBT, the risk of death due to CRC decreased depending on the number of FOBTs. There was a 44% HR reduction in CRC mortality for subjects frequently undergoing FOBT. A Cochrane systematic review on CRC screening using FOBT, which combined four RCTs, showed a 16% reduction in the relative risk of CRC mortality. Furthermore, when adjusted for screening attendance in the individual studies, there was a 25% relative risk reduction.^12^ Three Japanese case-control studies evaluated the efficacy of FOBT and found significant reductions in CRC mortality, ranging from 23% to 81%.^13^^–^^16^ According to the International Agency for Research on Cancer report,^17^ the relative risk of death due to CRC was 10–40% lower among subjects who had undergone FOBT screening than among those who had not in three cohort studies.^18^^–^^20^

In the analysis of screening colonoscopy, there was a 69% reduction in the risk of death due to CRC for subjects undergoing screening colonoscopy at the start of follow-up. Although the number of subjects who underwent screening colonoscopy was small and our finding may have been a chance finding, our study suggested that the effectiveness of screening colonoscopy in reducing the risk of CRC mortality and incidence tend to be attenuated after >10 years. This result might support the recommendation of many medical organizations that colonoscopy should be performed every 10 years for CRC screening. In Japan, colonoscopy is the opportune screening for CRC; however, its interval is not determined.^5^ A meta-analysis on observational studies and a 22-year follow-up study showed that colonoscopy screening was associated with a 68% reduction in CRC mortality.^21^^,^^22^ Our results were consistent with the results of these studies.

In the analysis of FOBT, the incidence of CRC also decreased depending on the number of FOBTs. This was prominent in advanced CRC; however, there was no significant reduction in the risk of non-advanced CRC. In the analysis of screening colonoscopy, subjects undergoing screening colonoscopy at the start of follow-up had a reduced incidence of advanced and non-advanced CRC.

It is possible that colonoscopy is able to detect abnormalities from the stage of adenoma, and adenoma removal reduces the subsequent risk of both advanced and non-advanced CRC. This is consistent with the findings of a previous cohort study.^22^

However, considering that sensitivity of FOBT is lower than that of colonoscopy for detecting adenomas, many adenomas might be undetected and untreated during screening with FOBT. A meta-analysis showed that the average sensitivity of FOBT for CRC and advanced neoplasia (defined as CRC, adenomas ≥10 mm, or adenomas with ≥25% villous component and/or high-grade dysplasia) were 93% (95% CI, 53–99%), and 48% (95% CI, 39–57%), respectively.^23^ FOBT cannot detect as many adenomas as colonoscopy.^24^ There are some cases where FOBT detects abnormality at the stage of adenoma; however, there must be many cases where FOBT detects abnormality at the stage of non-advanced CRC.^25^^,^^26^ Detection of non-advanced CRC does not reduce the number of non-advanced CRC, but it reduces the number of future advanced CRC. It was considered that there was a balance between the decrease in the number of future non-advanced CRC through partial detection of adenomas and the increase in the number of non-advanced CRC through the detection of non-advanced CRC during the 14-year follow-up. Hence, FOBT reduced the subsequent risk of advanced CRC and did not reduce the subsequent risk of non-advanced CRC in our study.

A previous RCT showed that there was a 20% and 17% relative risk reduction of CRC incidence for those who received annual FOBT screenings and biennial FOBT screenings, respectively.^27^ A meta-analysis of observational studies showed that colonoscopy screening reduced the incidence of CRC by 69%.^21^ In our study, the HRs of CRC incidence for subjects undergoing FOBT twice or thrice and screening colonoscopy at the start of follow-up reduced by 32% and 60%, respectively. This difference may be due to the difference in non-advanced CRC incidence.

The effectiveness of FOBT on CRC mortality and incidence was greater for the subjects screened twice or thrice than for those screened once. Although multiple FOBTs might increase the likelihood of false positives and the possibility of undergoing subsequent diagnostic colonoscopy, our findings reinforce the importance of undergoing FOBT every year as recommended by many medical organizations.

For the analysis of FOBT, we found a small reduction in the risks of all-cause mortality excluding CRC and death from all cancers excluding CRC but no reduction in the risk of all cancer incidence excluding CRC in the screened subjects. This result might indicate that the screened subjects were less likely to die and that there was a little selection bias. However, the degree of mortality reduction of CRC was greater than that of all causes of death excluding CRC. Therefore, FOBT was considered to reduce death due to CRC. For the analysis of screening colonoscopy, we did not find differences in all-cause mortality excluding CRC or all cancer incidence excluding CRC between screened subjects and unscreened subjects.

In this study, Q10 was used as the start of follow-up. The exclusion of cases with CRC during Q00–Q10 might distort the results. In a sensitive analysis, we conducted an analysis of FOBT including the incidence of CRC from Q00 to Q10. The HRs of death (eTable 2) were similar with those of Table 3. Meanwhile, colonoscopy at Q10 for cases with CRC detected during Q00–Q10 would very likely to be conducted as follow-up colonoscopy, not as screening colonoscopy. Therefore, we believe the analysis of colonoscopy, including the incidence of CRC from Q00 to Q10, was not our main topic. Concerning the risk of CRC incidence, if cancers whose symptoms appear after the start of follow-up were detected via cancer screening before the start of follow-up, these cases were excluded only in the screened group, which leads to the overestimation of the effect of screening on reducing the incidence of CRC. On the other hand, if cancers whose symptoms appear after the end of the follow-up were detected via cancer screening before the end of the follow-up, these cases were included only in the screened group, which leads to the underestimation of the effect of screening on reducing the incidence of CRC. If those who had frequently undergone CRC screenings from Q00 to Q10 underwent CRC screenings frequently in the follow-up period and those who had seldom undergone CRC screenings from Q00 to Q10 seldom underwent CRC screenings in the follow-up period, the overestimation and underestimation of the effect of screening on reducing the incidence of CRC might be offset.

The strengths of this study are as follows. This was a large population-based prospective study with a long follow-up period and a high response rate. We used multiple screening histories of FOBT and colonoscopy. This prospective study enables the assessment of all causes of death excluding CRC and all cancer incidence excluding CRC and is the first prospective cohort study to assess the effectiveness of FOBT and screening colonoscopy simultaneously using multiple screening histories.

This study has several limitations. First, we determined the screened groups based on self-reports. There might lead to misclassification, resulting in an underestimation of the screening effectiveness. Case-control studies usually identify screening histories by reviewing the record of the subjects kept at the Screening Center. This method limits misclassification; however, it cannot cover the screening histories of subjects outside of the center. Those can be covered by self-reports, which is an advantage of this study. Second, we assumed that subjects who underwent both colonoscopy and FOBT in the same year underwent colonoscopy because of a positive FOBT result and that this colonoscopy was the detailed examination and not screening colonoscopy. However, some patients might have undergone colonoscopy first or both colonoscopy and FOBT in the same year, even if the FOBT result was negative. In addition, a subjects might have undergone both FOBT and sigmoidoscopy or colonoscopy intentionally at the same time for medical checkup. In such cases, colonoscopy should be classified as screening colonoscopy, not as diagnostic colonoscopy, and they should be included in the analysis of screening colonoscopy, not in the analysis of FOBT. We repeated the analysis of FOBT after excluding subjects who underwent both colonoscopy and FOBT in the same year and the analysis of screening colonoscopy after including them. The HRs of CRC death and incidence of the screened subjects increased slightly, and the HRs of all causes of death excluding CRC did not change (data not shown). Third, there was no information on the quality of colonoscopy in this study, and we could not distinguish between total colonoscopy and sigmoidoscopy. However, even if patients underwent sigmoidoscopy at a medical checkup, it is often performed together with a FOBT in Japan.^28^ In our study, we defined screening colonoscopy as that performed without FOBT; therefore, screening colonoscopy is considered almost complete colonoscopy. Fourth, there might be unmeasured confounding factors, such as healthy lifestyle and health-consciousness, influencing the relationship between undergoing screenings for CRC and the mortality and incidence of CRC. Fifth, we could not distinguish between guaiac-based FOBT and FIT in this study. However, almost all FOBTs are performed using FIT in Japan,^5^ and the analysis of FOBT is considered to show the effect of FIT. Finally, we excluded subjects who did not respond at Q05 or Q10, which might lead to selection bias.

In conclusion, our study confirmed that depending on frequency of FOBT and recency of colonoscopy, they reduced the risk of death due to CRC and the incidence of CRC.

## SUPPLEMENTARY MATERIAL

eTable 1. Hazard ratio and 95% confidence interval for the recency of FOBT and subsequent risk of colorectal cancer in 27,974 subjects in the JPHC Study

eTable 2. Hazard ratio and 95% confidence interval for the number of FOBTs and subsequent risk of colorectal cancer in 28,126 subjects in the JPHC Study, including incident cases during Q00 ∼ Q10
