# Supplementary material for: Effectiveness of Screening Using Fecal Occult Blood Testing and Colonoscopy on the Risk of Colorectal Cancer: The Japan Public Health Center-based Prospective Study
Source: J Epidemiol. 2023 Feb 5;33(2):91–100. doi: 10.2188/jea.JE20210057 (PMC9794451; doi:10.2188/jea.JE20210057)
Supplement: Supplementary file 1 [file je-33-091-s001.pdf]

eTable 1. Hazard ratio and 95% confidence interval for the recency of FOBT and subsequent risk of colorectal cancer in 27,974 subjects in the JPHC Study

	The recency of FOBT								
	Unscreened	10 years before the start of follow-up		5 years before the start of follow-up		The start of follow-up		*P* for trend
Death								
Person-years of follow-up	162,124.6	14,287.4		46,520.4		149,872.2		
Colorectal cancer								
Number of deaths (n=168)	104	7		22		35		
HR (95% CI)[b]	1.00 (reference)	0.70	(0.32–1.51)	0.73	(0.46–1.17)	0.40	(0.27–0.60)	<0.01
HR (95% CI)[c]	1.00 (reference)	0.89	(0.41–1.93)	0.98	(0.60–1.61)	0.52	(0.33–0.82)	<0.01
All cancers excluding colorectal cancer								
Number of deaths (n=1,161)	606	62		135		358		
HR (95% CI)[b]	1.00 (reference)	1.05	(0.81–1.36)	0.77	(0.64–0.93)	0.69	(0.60–0.79)	<0.01
HR (95% CI)[c]	1.00 (reference)	1.11	(0.85–1.45)	0.85	(0.70–1.04)	0.78	(0.67–0.91)	<0.01
All causes of death excluding colorectal cancer								
Number of deaths (n=3,191)	1,683	168		412		928		
HR (95% CI)[b]	1.00 (reference)	0.98	(0.84–1.15)	0.85	(0.76–0.94)	0.67	(0.61–0.72)	<0.01
HR (95% CI)[c]	1.00 (reference)	1.04	(0.89–1.23)	0.95	(0.84–1.06)	0.77	(0.70–0.85)	<0.01
Incidence								
Person-years of follow-up	157,735.6	13,928.1		45,375.5		146,763.9		
Colorectal cancer								
Number of cases (n=800)	409	33		100		258		
HR (95% CI)[a]	1.00 (reference)	0.89	(0.63–1.28)	0.84	(0.67–1.05)	0.69	(0.58–0.81)	<0.01
HR (95% CI)[b]	1.00 (reference)	0.95	(0.66–1.37)	0.90	(0.71–1.14)	0.72	(0.60–0.87)	<0.01
Advanced colorectal cancer								
Number of cases (n=410)	236	12		47		115		
HR (95% CI)[a]	1.00 (reference)	0.52	(0.29–0.93)	0.66	(0.48–0.90)	0.50	(0.40–0.63)	<0.01
HR (95% CI)[b]	1.00 (reference)	0.60	(0.33–1.08)	0.76	(0.55–1.06)	0.58	(0.44–0.75)	<0.01
Non-advanced colorectal cancer								
Number of cases (n=307)	130	15		45		117		
HR (95% CI)[a]	1.00 (reference)	1.31	(0.77–2.25)	1.23	(0.87–1.73)	0.99	(0.77–1.29)	0.94
HR (95% CI)[b]	1.00 (reference)	1.29	(0.75–2.22)	1.20	(0.84–1.74)	0.96	(0.71–1.29)	0.81
All cancers excluding colorectal cancer								
Person-years of follow-up	155,206.7	13,579.2		44,188.1		142,408.3		
Number of cases (n=3,087)	1,305	139		398		1,245		
HR (95% CI)[a]	1.00 (reference)	1.09	(0.91–1.30)	1.04	(0.93–1.16)	1.05	(0.97–1.13)	0.29
HR (95% CI)[b]	1.00 (reference)	1.08	(0.90–1.29)	1.03	(0.91–1.16)	1.03	(0.94–1.13)	0.55

CI, confidence interval; FOBT, fecal occult blood test; HR, hazard ratio.

[a] Adjusted for age at Q10, sex, study area, smoking status, alcohol drinking status, history of diabetes, occupation, body mass index, physical activity, red and processed meat intake, vegetable intake, fish intake, fruit intake, diary intake, and coffee intake

[b] Additionally adjusted for the number of chest and stomach X-rays

eTable 2. Hazard ratio and 95% confidence interval for the number of FOBTs and subsequent risk of colorectal cancer in 28,126 subjects in the JPHC Study, including incident cases during Q00 ~ Q10

	Number of FOBTs					
	0	1		2 or 3		*P* for trend
Death						
Person-years of follow-up	162,482.2	104,748.7		107,370.6		
Colorectal cancer						
Number of deaths (n=180)	110	41		29		
HR (95% CI)[a]	1.00 (reference)	0.59	(0.41–0.85)	0.43	(0.28–0.67)	<0.01
HR (95% CI)[b]	1.00 (reference)	0.75	(0.51–1.11)	0.57	(0.35–0.94)	0.02
All cancers excluding colorectal cancer						
Number of deaths (n=1,175)	609	321		245		
HR (95% CI)[a]	1.00 (reference)	0.82	(0.72–0.94)	0.65	(0.56–0.76)	<0.01
HR (95% CI)[b]	1.00 (reference)	0.89	(0.77–1.02)	0.75	(0.63–0.90)	<0.01
All causes of death excluding colorectal cancer						
Number of deaths (n=3,221)	1,693	877		651		
HR (95% CI)[a]	1.00 (reference)	0.82	(0.76–0.89)	0.64	(0.58–0.70)	<0.01
HR (95% CI)[b]	1.00 (reference)	0.89	(0.82–0.98)	0.77	(0.69–0.86)	<0.01

CI, confidence interval; FOBT, fecal occult blood test; HR, hazard ratio.

[a] Adjusted for age at Q10, sex, study area, smoking status, alcohol drinking status, history of diabetes, occupation, body mass index, physical activity, red and processed meat intake, vegetable intake, fish intake, fruit intake, diary intake, and coffee intake

[b] Additionally adjusted for the number of chest and stomach X-rays
